# Targeting STAT-3 signaling pathway in cancer for development of novel drugs: Advancements and challenges

**DOI:** 10.1590/1678-4685-GMB-2018-0160

**Published:** 2020-02-10

**Authors:** Sundas Arshad, Muhammad Naveed, Mahad Ullia, Khadija Javed, Ayesha Butt, Masooma Khawar, Fazeeha Amjad

**Affiliations:** 1 University of Lahore, Department of Allied Health Sciences, Gujrat Campus, Pakistan.; 2 University of Central Punjab, Faculty of life sciences, Department of Biotechnology, Lahore, Pakistan.; 3 University of Gujrat, Department of Biochemistry and Biotechnology Sialkot sub Campus, Pakistan.

**Keywords:** STAT-3, DNA binding domain, apoptosis, drug discovery STAT-3 inhibitors

## Abstract

Signal transducers and activators of transcription 3 (STAT-3) is a transcription factor that regulates the gene expression of several target genes. These factors are activated by the binding of cytokines and growth factors with STAT-3 specific receptors on cell membrane. Few years ago, STAT-3 was considered an acute phase response element having several cellular functions such as inflammation, cell survival, invasion, metastasis and proliferation, genetic alteration, and angiogenesis. STAT-3 is activated by several types of inflammatory cytokines, carcinogens, viruses, growth factors, and oncogenes. Thus, the STAT3 pathway is a potential target for cancer therapeutics. Abnormal STAT-3 activity in tumor development and cellular transformation can be targeted by several genomic and pharmacological methodologies. An extensive review of the literature has been conducted to emphasize the role of STAT-3 as a unique cancer drug target. This review article discusses in detail the wide range of STAT-3 inhibitors that show antitumor effects both *in vitro* and *in vivo*. Thus, targeting constitutive STAT-3 signaling is a remarkable therapeutic methodology for tumor progression. Finally, current limitations, trials and future perspectives of STAT-3 inhibitors are also critically discussed.

## Introduction

**Figure 1:**
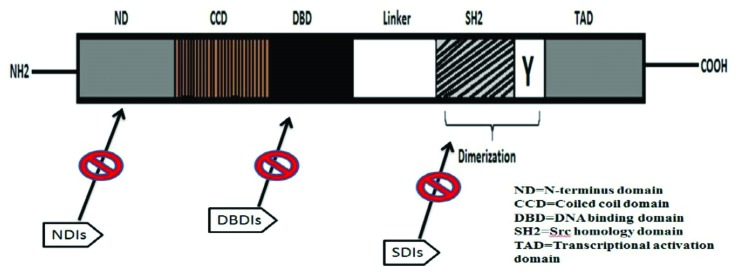
STAT-3 protein is segmental in organization. It contains an N-terminus domain (ND), coiled-coil domain (CCD), DNA-binding domain (DBD), Src homology 2 (SH2) domain, linker domain, Tyr (Y) residue, and the transactivation domain (TAD).

In 1994, STAT-3 was identified as a DNA transcription factor, bound with interleukin-6 responsive element in the promoter region of hepatic acute phase genes in response to IL-6 (Akira *et al.*, 1994). Moreover, it was identified as a DNA binding protein that was found to be expressed in response to epidermal growth factor response (Zhong *et al.*, 1994). The specific gene for encoding STAT-3 is located on the long arm of chromosome 17 at position 21 (17q21). This specific gene encodes a protein made up of 770 amino acids having molecular weight of up to 92 kDa. This protein structure can be divided into DNA binding domain, coiled coil domain (CCD), N-terminus domain (NTD), C-terminal domain (CTD) also called transactivation domain, and SH2 domain (Figure 1).

Serine and tyrosine are present at residues 727 and 705 positions respectively in cytosolic C-terminal or transactivation domain. These residues undergo phosphorylation after STAT-3 activation by cytokines and growth factors constituting Epithelial Growth Factor (EGF) (Cao *et al.*, 1996), Platelet-Derived Growth Factors (PDGF) (Vignais *et al.*, 1996), IL-6 (Akira *et al.*, 1994) that will also stimulate the cytosolic proto-oncogenes and tyrosine protein kinases like Src (Yu *et al.*, 1995), and Ras protein (Giordano *et al.*, 1997; Aggarwal *et al.*, 2009). Moreover, various other carcinogens have been recognized to initiate the expression of STAT-3 like cigarette smoke (Arredondo *et al.*, 2006), polychlorinated biphenyls, and 7,12-dimethylbenz[a]anthracene(DMBA) (Tharappel *et al.*, 2002; Chan *et al.*, 2004).

## STAT-3 signaling via IL6α receptor and Gp 130 subunit

Cancer progression and inflammatory responses are associated with cytokines activity mediated by STAT-3. The activation of STAT-3 is also carried out by other plasma membrane receptors, like tyrosine kinases comprising EGF and c-Met (Boccaccio *et al.*, 1998; Gao *et al.*, 2007; Quesnelle *et al.*, 2007). Two of the functionally most important families of cytokines are IL6 and IL10. Glycoprotein 130 (Gp 130) is a general receptor subunit for interleukin-6 family of cytokines. Other ligands include cardiotrophin-1 (CT-1), ciliary neurotrophic factor (CNTF), IL-11, leukemia inhibitory factor (LIF), and oncostatin M (OSM), (Hibi *et al.*, 1990; Hirano *et al.*, 1997). Gp 130 arbitrates signals that are important in the immune, nervous, hematopoietic, cardiovascular and endocrine systems, bone metabolism, inflammation, plasmacytoma genesis, acute phase response, liver regeneration, osteoporosis, and hepatocyte maturation (Yamasaki *et al.*, 1988; Suematsu *et al.*, 1989, 1992; Lord *et al.*, 1991; Brem and Thoenen, 1993; Escary *et al.*, 1993; Kopf *et al.*, 1994, 1998; Ramsay *et al.*, 1994; Hilbert *et al.*, 1995; Cressman *et al.*, 1996; Romani *et al.*, 1996; Yoshida *et al.*, 1996; Kumanogoh *et al.*, 1997; Betz *et al.*, 1998; Hirota *et al.*, 1999; Kamiya *et al.*, 1999; Ohtani *et al.*, 2000).

**Figure 2:**
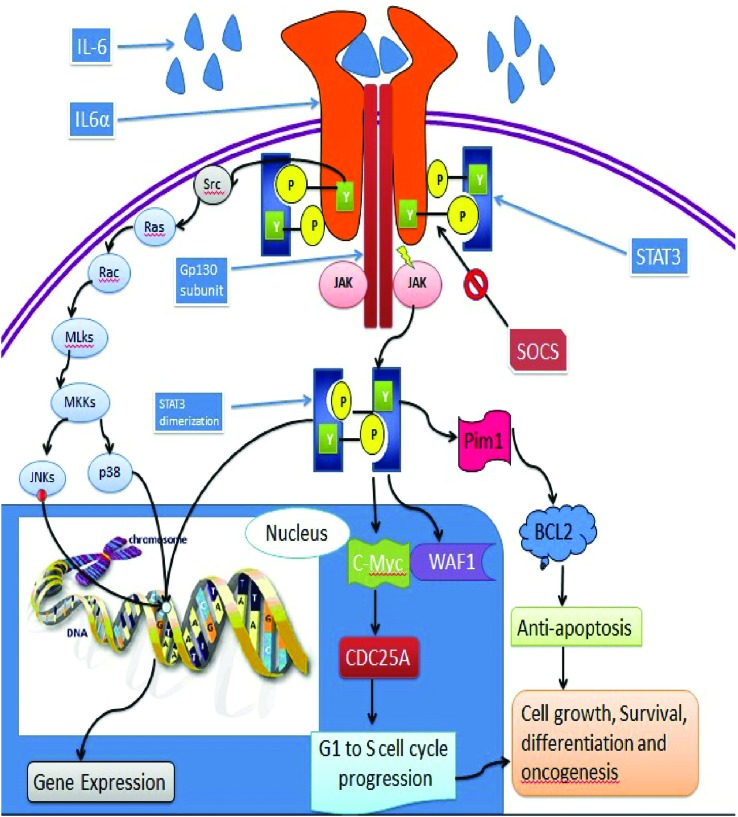
Polypeptides bind to their associated receptors and trigger the tyrosine (Tyr) kinase (TK) events of the receptors, Src or JAKs. Underlying STAT-3 action is the stimulated receptor for phosphorylation on specific Tyr residue by TKs that causes STAT-3:STAT-3 dimerization and stimulation. STAT-3 mounts up in the nucleus, where it binds to specific DNA response elements in the promoters of target genes, which causes gene transcription.

Activation of Gp 130 homo-dimerization is done by binding of IL6 and IL11 to IL6Rα and IL11Rα receptor subunits respectively, while activation of hetero-dimeric Gp 130 receptor complexes is done by some of the IL-6 family ligands (consisting of CNTF, LIF, CT-1, IL27, and oncostatin M) (Table 1) (Lacy *et al.*, 2007). Binding of Gp 130 with ligands activates the cytosolic kinases, like Janus kinases (Jak1, Jak2, and Tyk2) (Guschin *et al.*, 1995; Ernst *et al.*, 1996) and cause the phosphorylation of tyrosine residues in the cytoplasmic region of the cytokine receptor (Figure 2). The activation of STAT-3 also requires cytoplasmic tail of Gp 130 having four distal membrane residues while its regulation requires binding of activated Gp 130 with SOCS3 that results in its proteosomal degradation.

The activated growth factor receptors Janus kinases (JAKs) or Src tyrosine kinase cause activation of STAT3 that begins with the phosphorylation of a critical tyrosine residue (Tyr705) on its Src homology 2 (SH2) domain. Upon activation, STAT3 forms dimers through a reciprocal phosphotyrosine (pTyr705):SH2 domain interaction that translocate to the nucleus where the dimers bind to the promoters of target genes and activate specific gene expression. The activated JAKs phosphorylate serine residues of the STAT-3 at position 727. Transcriptional activity of STAT-3 is modulated by phosphorylation of serine residue at position 727 of many proteins like protein kinases C (PKC) (Jain *et al.*, 1999), CDK5, and mitogen activated protein kinases (Fu *et al.*, 2004). PKC-ε binds with the STAT-3 and phosphorylates its serine residue at position 727, which enhances its transcriptional activity (Wen and Darnell, 1997; Yokogami *et al.*, 2000; Aziz *et al.*, 2007; Yue and Turkson, 2009).

Various types of post-translational modifications, besides Y705 phosphorylation, are required for the activation of STAT-3. S727 phosphorylation of STAT-3 by extracellular signal regulated kinase 1 and 2 (ERK1/2) induces full activation of STAT-3 (Couto *et al.*, 2012). Dimerization of STAT-3 is mediated by a histone acetyltransferase p300 by acetylation at position K685, which is reversible by the type I histone deacetylase (HDAC) (Plaza-Menacho *et al.*, 2007). K180 methylation of STAT-3 is also required for its activation, mediated by a lysine methyltransferase EZH2 included in the polycomb repressive complex 2 (PRC2) (Yuan *et al.*, 2005).

## Inhibition of STAT-3

There are several mechanisms that negatively regulate STAT-3 activity by the protein inhibitor of activated STAT (PIAS), suppressors of cytokine signaling (SOCS), ubiquitination dependent proteosomal degradation, and protein phosphatases (Table 1) (Stahl *et al.*, 1995; Chung *et al.*, 1997; Starr *et al.*, 1997; Daino *et al.*, 2000)

**Table 1:** Activators and inhibitors of STAT-3.

Activators				Inhibitors		
Cytokines	Growth factors	Carcinogens	Others	Synthetic	Natural	Others
Cardiotrophin-1	EGF	Diesel & exhaust particles	Bile acids	AG 490	Caffeic acid	EKB569
IFN-γ	PDGF	Tobacco	Diazoxide	Aurothiomalate	Chalcone	Rituximab
IL-6	TGF-α	UVB	Genistein3	BMS-354825	Cucurbitacin	GQ-ODN
IL-10	G-CSF	HCV	Isoliquiritigenin	CADPE	Emodin	TKS –050
IL-11	GM- CSF	LPS	Leptin	Ethanol	Genistein	STA21
IL-21	RUNX1	RSV	Morphine sulfate	PDP	Indirubin	Retinoic acid
IL-27	ISRE	HPV	Olanzapine	PS-341	Parthenolide	TGF-β1
LIGHT	CRE	CNTF	Black soy peptides	S31-M2001	Piceatannol	17β-estradiol
MIP-1α	FGF1	LIF	CaMKIIg Acid	Sodium	Resveratrol	COX-2
RANTES	FGF2		Sphingomyelinase Acid	Statin	Capsaicin	
SLF25	IGF-IR		Ceramidase	T40214	CDDO-Me	
MCP-1	CSF1R		Colivelin	Atiprimod	Curcumin	
CNTF	AgtR2		Ruxolitinib Phosphate	Auranofin	EGCG	
IL-5	GPCRs			Stattic	Flavopiridol	
IL-9				Dobesilate	Ursolic acid	
IL-12				Platinum compounds	Guggulsterone	
IL-22				Y(p)LPQTV	Magnolol	
TNF-α						

### Inhibition by SOCS

Blocking of subsequent signaling, which requires phosphorylation and activation of STAT-3, is carried out by the binding of SOCS proteins with the JAK activation loop having SH2 domain (Zhang *et al.*, 1999). Until now, there are eight different SOCS proteins that are identified with same structures (Yoshimura *et al.*, 2007). In mouse skin wound healing, SOCS-3 modulates the Gp 130-STAT-3 signaling pathway, which shows that STAT-3 is essential for wound healing (Zhu *et al.*, 2008). Similarly, tissue-specific SOCS3 aberrations in mice upregulate the signaling of ligand-dependent Gp 130, and substituting Y757F tyrosine-to-phenylalanine in the respective *Gp 130*Y757F knock-in mutant mice and results in hyperactivation of STAT-3 and STAT-1 (Tebbutt *et al.*, 2002; Jenkins *et al.*, 2005).

STAT-1 and STAT-3 are able to regulate each other in the framework of Gp 130 mediated STAT activation (Ernst *et al.*, 2008; Musteanu *et al.*, 2010). Likewise, in normal macrophages, inflammatory responses are generated when STAT-3 is stimulated by binding of IL-6 (Unver *et al.*, 2018). However, in Gp 130 Y757F mutant macrophages, the induction of transcriptional repressor can inhibit inflammatory gene responses in STAT-3 signaling by IL-6 (El Kasmi *et al.*, 2007; Murray, 2007). Therefore, an efficient anti-inflammatory feedback can be stimulated with continuous Gp 130 and STAT-3 activation in SOCS3-deficient macrophages (Croker *et al.*, 2003; Lang *et al.*, 2003; Yasukawa *et al.*, 2003; Jenkins *et al.*, 2005).

### Inhibition by PIAS-3 and other deregulators

Another mechanism to inhibit the STAT-3 regulation is PIAS. PIAS-3 inhibits the process of transcription by collaborating with phosphorylated STAT-3 (Chung *et al.*, 1997) that is produced by attachment of phosphate groups during normal regulation (Saini *et al.*, 2018). In pancreatic cancerous cells, Smad4 inhibits the tyrosine phosphorylation of STAT-3 (Zhao *et al.*, 2008). Activation of STAT-3 is also inhibited by various protein tyrosine phosphatases, comprising CD-45 (Irie-Sasaki *et al.*, 2001), PTEN (Sun and Steinberg, 2002), SHP-1 (Migone *et al.*, 1998), and SHP-2 (Schaper *et al.*, 1998)

### Inhibition via ubiquitin degradation

Another pathway to inhibit STAT-3 regulation is the ubiquitin-proteasome pathway. This pathway is essential for depletion of different transitory cellular proteins. It also controls the regulatory mechanism of cellular processes. STAT-3 degradation occurs through this pathway (Daino *et al.*, 2000; Perry *et al.*, 2004; Ulane *et al.*, 2005). In IL-6-dependent KT-3 cells, proteosomal degradation of STAT3 occurs when it binds with the biotinylated ubiquitin, without effecting the expression of STAT1 and STAT5 (Daino *et al.*, 2000). Moreover, caspases also inhibit STAT-3 (Darnowski *et al.*, 2006). STAT-3 signaling inhibition is due to reduction in STAT-3 binding with DNA, STAT-3-driven reporter protein (luciferase) activity, STAT-3-dependent genes, and due to increased sensitivity to apoptotic stimuli.

STAT-3 is essential for many interconnected signaling pathways. Enhanced STAT-3 activity in cancer can be due to excess of growth factors and IL6-family cytokines in the cancer microenvironment. STAT-3 activation or secretion of inflammatory agents occurs by proto-oncogenes activation, tumor-suppressor genes, chromosomal rearrangement, and other genomic alterations in tumor cells. Unexpectedly, there is no inherent confirmation for triggering the mutations in STAT*-*3 itself. However, in many cases, frame deletion mutations in Gp 130 and point mutations in Jak2 (Morgan and Gilliland, 2008) promote the ligand-independent activation of STAT-3 found in hepatocellular carcinomas (Rebouissou *et al.*, 2008). Under biological conditions, the stimulation of STAT-3 affects several inhibitory proteins, which also deregulate STAT-3 activity (Greenhalgh and Hilton, 2001). The abnormality in STAT-3 causes embryonic lethality, (Takeda *et al.*, 1997) and tissue-specific abnormality that causes the destruction of liver cells (Niu *et al.*, 2002), macrophages (Takeda *et al.*, 1999), keratinocytes (Sano *et al.*, 1999) and mammary or thymic epithelial cells (Chapman *et al.*, 1999).

## Different functions of STAT-3

### Inflammation

STAT-3 is a mediator of inflammation, suggested by various lines of evidences (Pfitzner *et al.*, 2004). Initially, STAT-3 was revealed as an acute-phase response protein, due to its inflammatory responses. Second, STAT-3 is activated mainly by pro-inflammatory agents, such as IL-6 that is a most important mediator of inflammation in the STAT-3 pathway (Zhong *et al.*, 1994). Likewise, cigarette smoke, tumor promoters, and lipopolysaccharides also can trigger the STAT-3 pathway (Kobierski *et al.*, 2000; Arredondo *et al.*, 2006). Third, binding of acute-phase proteins on DNA promoter region compete with NF-κB, alternative pro-inflammatory transcription factor (Zhang and Fuller, 1997). Fourth, in accessory cells, NF-κB binding to the IL-12p40 promoter is controlled by STAT-3 (Hoentjen *et al.*, 2005). Fifth, oncogenic transformation via IL-11 and its Gp 130 receptor in inflammation-associated gastric epithelial cell is triggered by and dependent on enhanced expression of STAT-3 (Ernst *et al.*, 2008). Sixth, in various cell types IL-6-triggered STAT-3 activation has been shown to be dependent on cyclooxygenase 2 (Dalwadi *et al.*, 2005). All this verification supports the function of the STAT-3 pathway in inflammation.

### Transformation of cells

The stimulation of STAT-3 is mediated by various oncogenes having direct or indirect effects of STAT-3, protein tyrosine kinases, and viruses if transformed in to cells (Frank, 2007) proceeded by Src protein kinase (Yu *et al.*, 1995; Bromberg *et al.*, 1998). Similarly, in case of gastric cancer, the activation of STAT-3 by human T-cell lymphotropic virus I can transform T cells, involving a direct effects of STAT-3 and epithelial-mesenchymal transition (EMT). While the activation of cells in the microenvironment is dependent on indirect effects of STAT-3 (Migone *et al.*, 1995). Activation of STAT-3 is triggered by polyoma virus middle T antigen (v-Fps) that triggers Src family kinases, or activation is triggered by v-Sis, acting as a ligand for PDGF-R (Garcia *et al.*, 1997). STAT-3 signaling is also required for hepatocyte growth factor-Met mediated tumor genes factor. Cell growth in soft agar, cell transformation, and tumors in nude mice are stimulated by STAT-3. The activated form of STAT-3 detected in tumors confirms that it is an oncogene (Bromberg *et al.*, 1999).

### Apoptosis suppression

The activation of STAT-3 can be triggered by oncogenic transformation of the cells responsible for the survival signal. Conditional inactivation of STAT-3 has pro-apoptotic functions during mammary gland involution (Chapman *et al.*, 1999). In most cells, STAT-3 activation can be suppressed by apoptosis. The induction of these special effects appears due to several gene products that are synchronized by STAT-3. These include BCL-2, (Zushi *et al.*, 1998), survivin (Mahboubi *et al.*, 2001), BCL-XL (Catlett-Falcone *et al.*, 1999; Karni *et al.*, 1999), MCLl-1 (Karni *et al.*, 1999), CyclinD1 (Munoz *et al.*, 2014), and CLap2 (Bhattacharya and Schindler, 2003). Furthermore, tumor cells exhibiting constitutive activation of STAT-3 also express cell survival genes (Aoki *et al.*, 2003; Kanda *et al.*, 2004). Suppression of STAT-3 activation also suppress the expression of all cell survival gene products, that potentiate apoptosis (Konnikova *et al.*, 2003). Down-regulation of STAT-3 is promoted by apoptosis, leading to expression of FAS protein (Ivanov *et al.*, 2001).

### Cellular proliferation

STAT-3 activation can also be linked with proliferation of tumor cells, because it induces the expression of cyclin D1 (Masuda *et al.*, 2002). STAT-3 increases the expression of numerous growth-promoting genes, like pim-1 (Kiuchi *et al.*, 1999) and myc (Shirogane *et al.*, 1999). Other reports suggested that STAT-3 can downregulate the expression of cell cycle inhibitor p21(waf1), showing abnormal cell production and cell cycle succession (Bellido *et al.*, 1998) by means of p21 (Roninson, 2002). In cellular transformation, STAT-3, without varying the regulation of myc promoter, was found to impede the transcriptional stimulation of the p21 gene in the Akt pathway (Barré *et al.*, 2003). Two stages of polymorphism have also been found to be vital in producing a negative response and in causing certain types of tumors (Xin-hua and Rui-yu, 2012).

### Cellular invasion

Numerous reports indicated that STAT-3 activation plays the most significant function in tumor cell invasion, and its inhibition reduces invasion (Barré *et al.*, 2003; Ma *et al.*, 2007; Yakata *et al.*, 2007; Xiong *et al.*, 2008; Zhao *et al.*, 2008). The expression of matrix metalloproteinase (MMP)-2 and MMP-1 is regulated by STAT-3 activation that initiates tumor invasion and metastasis (Xie *et al.*, 2004; Itoh *et al.*, 2005). Direct interaction of STAT-3 with MMP-2 up-regulates the transcription of MMP-2. In highly metastatic cells, invasiveness of the tumor cells is suppressed by obstruction of activated STAT-3, while metastasis of cutaneous squamous cell carcinoma is associated with overexpression of phosphorylated STAT-3 (Suiqing *et al.*, 2005). Tissue inhibitors of metalloproteinase hinders the activity of metalloproteinases, which are upregulated by STAT-3, and decrease invasiveness in certain cancer cells (Dien *et al.*, 2006). The expression of the *MUC1* gene mediates tumor invasion and it is also controlled by STAT-3 (Gaemers *et al.*, 2001). Thus, STAT-3 tumor invasion can be induced through several mechanisms.

### Metastasis and angiogenesis

A STAT-3 link to angiogenesis was revealed by factor-induced angiogenetic action in chick chorioallantoic membrane stimulated from granulocyte-macrophage colony (Valdembri *et al.*, 2002). Tumor angiogenesis and Vascular Endothelial Growth Factor appearance is also due to STAT-3 upregulation (Niu *et al.*, 2002). Activated STAT-3 in the majority of cancer cells has an effect on vascular endothelial growth factor (VEGF) (Wei *et al.*, 2003a,b). Consequently, downregulation of STAT-3 can suppress the appearance of VEGF and reduce angiogenesis. Metastasis of human hepatocellular carcinoma and inhibition of growth was found by antisense oligonucleotide targeting of STAT-3 (Li *et al.*, 2006). STAT-3 activation was also associated with metastasis of human melanoma to brain (Xie *et al.*, 2006). Moreover, VEGF and TWIST, another mediator of tumor metastasis, were synchronized by STAT-3 in an *in-vivo* analysis, whereas chemoresistance, angiogenesis, and cell survival were induced in an *in-vitro* study (Cheng *et al.*, 2008)

### Carcinogenesis

The stages of carcinogenesis, such as tumor initiation and tumor progression, are enhanced by irregularities in the STAT-3 signaling pathway. Skin cancer can be suppressed by blocking aberrant expression of STAT-3 (Nagpal *et al.*, 2002; Chan *et al.*, 2004; Ahsan *et al.*, 2005). When 12-*O*-tetradecanolyphorbol-13-acetate was used as the promoter and 9,10- dimethylbenz-[a-]anthracene was used as an activator, development of skin tumor was entirely opposed in STAT-3-deficient mice (Dvorak *et al.*, 2007). The activation of STAT-3 is an early event in oral carcinogenesis by tobacco consumption (Jang *et al.*, 2008). The triggering of STAT-3 has also been associated with hepatocarcinogenesis (Ahn *et al.*, 2008).

### Radioresistance and chemoresistance

The aberrant activation of STAT3 in cancerous cells is also associated with chemo- and radioresistance (Real *et al.*, 2002; Bharti *et al.*, 2004; Boehm *et al.*, 2008). This resistance was shown by the up-regulation of anti-apoptotic genes activated by STAT-3 (Bhardwaj *et al.*, 2007). Therefore, it is suggested that chemoresistance can be stroked by modulating STAT-3 activation (Otero *et al.*, 2006). Several studies indicated that the deletion of STAT-3 causes B cells to become hyper responsive to irradiation (Drews, 2000). *In vivo* studies on gene-targeted mice showed that binding of IL-6 and IL-10 and some other BCR ligands to the receptors is associated with B1 cell radioresistance.

### Epithelial mesenchymal transition (EMT)

EMT is the binding of epithelial cells with mesenchymal cancer-associated fibroblasts (CAFs), which results in loss of adhesion among cells, causing tumor progression (Larue and Bellacosa, 2005). In cancers mediated by STAT-3, EMT gets involved in cancer progression by the IL-6 and JAK-STAT-3 pathway. In cases of gastric cancer, CAFs promote EMT in cells by secreting enough IL-6 that in turns activates the STAT-3 pathway. The minimization effect of IL-6 and use of AG490 inhibits STAT-3 that results in tumor metastasis induced by CAFs *in vivo*. CAFs are important in cancer progression by indirect inhibition of the JAK/STAT pathway in a microenvironment (Wu *et al.*, 2017a; Karakasheva *et al.*, 2018).

### Prevention of ROS production in mitochondria during stress

STAT-3 expression has a crucial role in cardiac protection against stresses. STAT-3 influences the activity of cytochrome I and II in mitochondria. The deletion of STAT-3 in mice cardiomyocytes was found to reduce the activity of complex I and II up to 50%. But in the case of overexpression of transcriptionally inactive STAT-3, the activity of both complexes was reduced only by 20%. Moreover, the overexpressed STAT-3 in mitochondria in comparison to wild type mitochondria showed protection from ischemia. Ischemia causes the production of reactive oxygen species (ROS) from complex I in case of wild type mitochondria, which delocalizes cytochrome c from the inner mitochondrial membrane and eventually results in its release from mitochondria causing apoptosis. On the contrary, overexpressed STAT-3 mitochondria blocked ROS production, preventing cytochrome c release and hence apoptosis (Szczepanek *et al.*, 2011, 2012).

### Embryonic development

OCT4 is a transcriptional factor responsible for the regulation of several genes, including NANOG and SOX2, and maintenance of embryonic stem cells pluripotency. STAT-3 is also expressed in the embryo and is involved in preserving the pluripotency of ESCs by interacting with OCT4 and NANOG through regulating klf4 (Do *et al.*, 2013). Elimination of STAT-3 in mouse embryonic cells showed the importance of STAT-3 in retaining inner cell mass (ICM) pluripotency, but the phosphorylation of STAT-3 by β-catenin and E cadherin is essential for this role. The blockage of E cadherin inhibits STAT-3 phosphorylation, disabling pluripotency preservation. Alternatively, N cadherin can also perform the E cadherin task (Hawkins *et al.*, 2012).

### Axonal degeneration

Degeneration of axons starts with changes in distal axon and presynaptic terminals, leading to irreparable damage and eventually death of brain cells. In axon regeneration, plasticity is maintained by neurotrophic factors released by the surrounding cells, especially Schwann cells. Ciliary neurotrophic factor (CNTF) and other neurotrophic factors are produced by Schwann cells for this function. CNTF activates STAT-3, which in turn interacts with stathmin. Stathmin is a protein that binds with the α/β-tubulin heterodimers, preventing the assembly of MT. STAT-3 resultantly promotes the regeneration of microtubules, preventing MT from destabilization, as studied in *pmn* mutant motoneurons (Selvaraj *et al.*, 2012).

## Strategies to target STAT-3 for novel cancer drugs

Understanding the mechanisms of STAT-3 activation and transcriptional events from the receptor on cell surface to the nucleus may provide different approaches to target STAT-3 in cancer therapeutics. Since the objective for drug discovery is to target a disease, it is essential to determine whether the proposed target can effectively bind with a specific drug. Drug affinity with a target depends on disease development, mode of action of the drug, and consequences of the approach of drug target. Research showed strong association of the location of the target relative to the disease (Gibbs, 2000; Turkson, 2004). It is well-known that STAT-3 plays a significant role as a principal regulator of biological and molecular actions. In addition, deregulation of STAT-3 leads to tumorigenesis (Yu and Jove, 2004; Darnell, 2005; Siddiquee *et al.*, 2007a). Therefore, targeting abnormal STAT-3 action by pharmacological or genetic approaches triggered apoptosis and growth arrest of tumor cells *in vitro* and tumor reversion *in vivo* (Darnell, 2005; Jing *et al.*, 2006; Leeman *et al.*, 2006; Siddiquee *et al.*, 2007b; Weerasinghe *et al.*, 2007). Actually, various studies showed STAT-3 as a promising cancer drug target, so a rationale can be developed for the discovery and design of anti-cancerous drugs (Table 2).

**Table 2:** Targets and Inhibitors of the STAT-3 signaling pathway.

Site of action	Class	Preclinical Data	Clinical Data	Challenges
Inhibitors of STAT-3 stimulation	**JAK1/2 inhibitor** Ruxolitinib	Tumor xenograft models	primary myelofibrosis (phase III)	Unfeasible in most cancers
	**Small-molecule JAK inhibitors** (LS-104, AG490, CEP701and ICNB18424)		(phase II)	Insignificant myelosuppression and a minor but recurrent GI toxicity
SH2 domain dimerization inhibitors	**Peptides**	Src-transformed	Nil	Poor cellular permeability in vivo stability
	(XpYL)	fibroblasts		
	**Peptidomimetics**	Breast, NSCLC		
	(ISS610)	Src-transformed		
		fibroblasts		
	**Small molecule**	Breast, sarcoma, Nil	Lack of potency and specificity	
	**inhibitor**	GBM		
	STA-21 and	GBM		
	analogues	Breast		
	LLL-3	Breast, HCC, GI		
	S3I-201	Breast		
	Static	CRC		
	OPB-31121	Liver		
	OPB-51602	Lung		
	**Natural**	RCC	Advanced hematologic and solid tumors (Phase I)	Unexpected toxicities Unpredictable PK properties
	**compounds**	Breast		
	Curcumin	Pancreas, HCC,		
	Curcumin	GI		
	analogues	NSCLC		
	(FLLL11, FLLL12, FLLL32)			
N terminal domain inhibitors	**Synthetic analogues for STAT-3 helix 2**	Inhibited cell growth and apoptosis of human MCF-7, MDA-MB-435 and MDA-MB-231 breast cancer cells		Peptides interaction with N-terminal domain and their mechanisms of action are still unknow
	**Synthetic peptide**			
	ST3-H2A2	Cause blockage of STAT-3 dimerization and apoptosis in prostate cancer cell lines		
Upstream tyrosine kinase inhibitors	**Small molecule**	EGFR NSCLC		Neurologic dose-limiting toxicities
	inhibitors	NSCLC	Phase I	Toxicity and lack of efficacy
	AZD1480	HNSCC	Phase II	Lack of potency and specificity
	(JAK1/2)			
	Dasatinib (Src)			
	**Natural**	HCC, breast,	Nil	
	**compounds**	pancreas		
	Butein Capsaicin	Prostate, gastric		
Inhibitors of Nuclear Translocation	Karyostatin		Nil	Inhibition of general trafficking through the nuclear membrane is probable to be harmful
	1ARajtadoneB	Human lung		
	Leptomycin	carcinoma cell		
	Bimax1, bimax2	lines		
		mouse		
		neuroblastoma		
		cell lines		
		HeLa cell lines		
STAT3 pathway oligonucleotides	**Antisense oligonucleotide**	Lymphoma	Phase I/II	Rapid degradation, not amenable to systemic administration
	AZD9150			
				
			Phase 0	
	**STAT3 decoy oligonucleotide**	NSCLC		
	(DNA-binding)	NSCLC, colorectal		
		glioma		
	**STAT3 post-transcriptional**	Breast, brain, SCC		
	(siRNA)			
STAT3 DNA-binding domain	**Platinum IV compounds**,	Breast		
		Colon	Nil	Lack of specificity
	IS3295,	NSCLC		
	CPA-1,			
	CPA-7,			

Abbreviations: RSV: Respiratory syncytial virus, ISRE: interferon-stimulated responsive element, CRE: cyclic AMP-responsive element, FGF: fibroblast growth factor, HPV: human papillomavirus infection, IGF-IR: insulin-like growth factor I receptor, VEGF: vascular endothelial growth factor, CNTF: Ciliary neurotrophic factor, LIF: Leukemia inhibitory factor, CSF1R: Colony Stimulating Factor-1 Receptor, GPCRs: G-Protein Coupled Receptors, : Angiotensin-II Receptor.

### Strategies to prevent STAT-3 stimulation

Targeting JAK/STAT signaling is the most used therapy in patients with myeloproliferative disorders, majority of which show the oncogenic*JAK2*V617F mutation. These gain-of-function mutations generate constitutive activation of JAK/STAT signaling, especially through STAT-3 and STAT-5. According to a phase III randomized-controlled study of the JAK1/2 inhibitor ruxolitinib, a prolonged survival was found with the inhibitor in comparison to the best available therapy in primary myelofibrosis (Harrison *et al.*, 2012). One way to prevent STAT-3 activation is to inhibit the tyrosine kinase events at receptor level, which is unfeasible in most cancers. (Jing and Tweardy, 2005; Quesnelle *et al.*, 2007; Boehm *et al.*, 2008; Wilks, 2008; Egloff and Grandis, 2009; Sen *et al.*, 2009a; Santos *et al.*, 2010; Verstovsek *et al.*, 2010).

Small-molecule JAK inhibitors (for example, LS-104, AG490, CEP701, and ICNB18424) have been tried in tumor xenograft models before several clinical trials (Sen *et al.*, 2009a; Santos *et al.*, 2010; Verstovsek *et al.*, 2010). Equally in *in vivo* and *in vitro*, AG490 inhibits the action of JAK2, decreases STAT-3 levels, stops STAT-3 DNA binding, and decreases leukemic cells growth (Fletcher *et al.*, 2009; Santos *et al.*, 2010). Its analog LS-104 was developed for acute lymphoblastic leukemia in a level II clinical trial (Santos *et al.*, 2010). INCB1824 suppresses phosphorylated STAT-3, which leads to V617F JAK2 gain-of-function mutation (Sen *et al.*, 2009a; Santos *et al.*, 2010). CEP-701, which is a JAK2 inhibitor, decreases the phosphorylation of STAT-3 in patients under therapy; however, its modest effectiveness in myelofibrosis patients is related with an insignificant myelosuppression and a minor but recurrent GI toxicity (Verstovsek *et al.*, 2010).

The comparatively modest feedback of agents targeting JAKs from cancer patients demonstrates that single pathways possibly will not satisfactory inhibit the activation of STAT-3. Unfortunately, early phase clinical trials of JAK1/2 inhibitors (e.g. AZD1480) and Src inhibitors (e.g. dasatinib) have revealed limited efficacy or excessive toxicity in advanced solid tumors (Murakami *et al.*, 2014). Possible explanations for toxicities and off-target adverse events are pathway redundancy and pathway cross-talk.

### Strategies to prevent protein–protein interaction

Targeting protein-protein interaction involves a scattered and huge surface area in contrast to the easily `druggable’ classic binding pocket found in receptor tyrosine kinases or other enzymatic targets (Fontaine *et al.*, 2015). Furthermore, STAT proteins share a highly homologous domain structure, making the specific targeting of STAT-3 more challenging. The main targets to prevent protein-protein interaction/dimerization in STAT-3 are SH2-domain and N-terminal domain, for which various drugs have been designed as discussed briefly below.

### Targeting the STAT-3 SH-2 domain

The first successful attempt at disrupting STAT-3:STAT-3 dimerization and its downstream transcription was the discovery of a phospho-peptide inhibitor (PY*LKTK), which is obtained from the STAT-3-SH2 domain-binding peptide sequence. However, the intrinsic pharmacokinetic properties of peptides, including poor cellular permeability and lack of stability*in vivo*, have curtailed their further development. Even second-generation peptidomimetics have failed to overcome these limitations (Turkson *et al.*, 2004).

Various approaches have been developed to inhibit protein-protein interaction. One of them is the use of pY-containing peptide. Researchers have produced pY-containing peptide structures to target the SH-2 domain of STAT-3, preventing recruitment of STAT-3 to stimulated receptors and homo-dimerization of STAT-3 (Germain and Frank, 2007; Quesnelle *et al.*, 2007; Fletcher *et al.*, 2009). The peptide containing pY residue was investigated to study the mechanism of STAT-3 inhibition for the first time. Inhibition activity of STAT-3 was based on the synthesis of a Y705 residue. This residue is phosphorylated, which promotes homodimerization of STAT-3, which in turn inhibits the binding of STAT-3 to the DNA *in vitro* (Table 3) (Germain and Frank, 2007; Fletcher *et al.*, 2009).

**Table 3:** Domains of STAT-3

Domain	Function
N-terminus domain	The N-terminus comprises a domain (ND) that facilitates STAT dimer–dimer communications in tetramer establishment that is vital to stabilize the dimers binding to DNA.
Coiled-coil domain	The coiled-coil domain (CCD) connects the (ND) N-terminus domain to the (DBD) DNA-binding domain and also take part in the communications with other proteins
The DNA-binding Domain	The DBD creates physical connection with SRE in target genes promoters and is associated to the Src homology 2 domains *via* the linker domain (Linker).
SH2 domain	The SH2 domain is central for dimerization of two STAT3 monomers.
Tyr (Y) residue	On phosphorylation of the specific Tyr (Y) residue in the transcriptional activation domain (TAD), the pTyr residue of one monomer and the Src homology 2 domains of a different monomer involve in shared pTyr-SH2 domain interaction
The transactivation domain	The transactivation domain assists the transcriptional stimulation of target genes and can comprise a serine residue essential for utmost transcriptional activity in the circumstance of some STAT proteins, including STAT3.

This methodology has been extended upon by a number of groups to take account of pY-containing peptide structures from further proteins that act together with SH-2 domains of STAT-3 (Germain and Frank, 2007; Fletcher *et al.*, 2009). pY-containing motifs with the SH-2 domain of STAT-3 have been explored. These motifs contain the four distinctive pY-containing (pYXXQ) motifs inside the signal-transducing subunits of Gp 130 of the activated IL-6 receptor complex with numerous pY-containing motifs after cytokine receptor chains (for example, IL-10 receptor, granulocyte colony-stimulating factor receptor, and leukemia inhibitory factor receptor) (Frank, 2007).

Unfortunately, peptide inhibitors have poor cell absorptive capacity and metabolic solidity, which encouraged a quest for small-molecule derivatives and peptidomimetics (Germain and Frank, 2007; Fletcher *et al.*, 2009). Numerous small-molecule inhibitors were identified by peptidomimetics and peptide-inspired coherent strategy together with *in silico* computational methodologies and *in vitro* high-output selection. In spite of developments in detecting small molecules and peptides that target the SH-2 domain of STAT-3, molecules with satisfying anticancer activity have yet to be revealed.

Another approach that blocks STAT-3-protein interactions via targeting SH-2 domain is using G-rich oligodeoxynucleotides, which produce potassium-dependent four-stranded assemblies to reside inside the SH-2 domains of STAT (Heinrich *et al.*, 2003; Germain and Frank, 2007; Quesnelle *et al.*, 2007; Fletcher *et al.*, 2009). G quartet oligodeoxynucleotides may interrupt the homo-dimerization of STAT-3 activity. The large size and potassium dependence of G quartet oligodeoxynucleotide make the cell less permeable, hence providing a great challenge for *in vivo* studies (Fletcher *et al.*, 2009).

Novel small molecular inhibitors that target the STAT-3-SH2 domain have been discovered through virtual screening and have demonstrated physiochemical properties, indicating their potential for clinical use. These constitute the largest class of STAT-3 inhibitors. Numerous preclinical studies have confirmed their mode of action and downstream effects on tumor cell inhibition in an array of animal models and cell lines. However, most of these compounds have yet to be explored in clinical studies due to concerns with their relative lack of potency and selectivity (Furqan *et al.*, 2013).

OPB-51602 and OPB-31121 are the only agents in this class to have reached early phase clinical trials in advanced solid malignancies. Although signals of efficacy were observed in tyrosine kinase inhibitor (TKI)-resistant EGFR-mutant NSCLC and gastrointestinal malignancies, further development of these compounds was limited because of the concerns about unpredictable pharmacokinetics profiles and potentially severe toxicities including lactic acidosis, peripheral neuropathy, and susceptibility to opportunistic infections (Wong *et al.*, 2015). A possible explanation for this unusual side effect profile is the ubiquitous expression of STAT-3 within the body and its diverse physiological roles, including modulation of mitochondrial metabolism and the immune system (Myers, 2009).

### Targeting STAT-3 N-terminal domain

**Figure 3:**
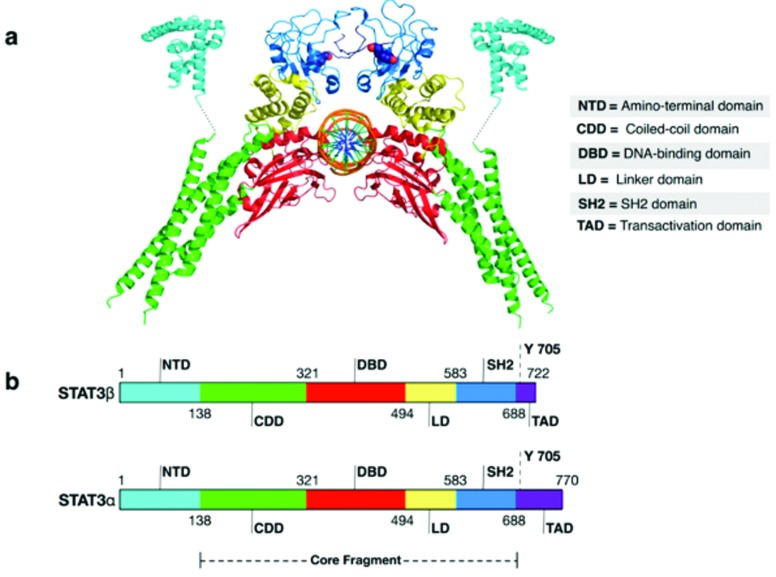
Three dimensional structure of STAT 3 indicating its active and inhibitory sites. (a) 4Z1A PDB hit of STAT 3. (b) Different domains of STAT 3 present in loops of STAT 3.

N-terminal domain is pivotal for the interaction of dimers of STAT-3 for the formation of tetramer, interaction with other regulators and the attachment of STAT-3 dimers to the sites of DNA, and protein interaction (Figure 3) (Sgrignani *et al.*, 2018). The N-terminal domain has a leading role in dimer formation, so it is a captivating approach to inhibit the function of STAT-3. The N-terminal domain of STAT-3 protein comprises eight helices with 130 amino acids (Blaskovich *et al.*, 2003; Timofeeva *et al.*, 2007). Studies found that synthetic analog of STAT-4’s helix 2 can target and disturb the structure of N-terminal. Based on the knowledge of the N-terminal domain of STAT-4, a synthetic analogue for STAT-3 helix 2 was designed that specifically binds to STAT-3 instead of binding with other members of STAT. As a result, transcriptional activity of STAT-3 is inhibited without affecting phosphorylation.

By fusing alpha helix 2 with ε-palmitoyl lysine and three residues of penetratin, a cell permeable form of the alpha helix was produced that retards cell growth and apoptosis of human MDA-MB-435, MCF-7, and MDA-MB-231 breast cancer cells. However, details of how these peptides interact with the N-terminal domain of STAT-3 and their mechanisms of action are still unknown (Timofeeva *et al.*, 2007). Another approach developed to inhibit dimerization via targeting the N-terminal domain is ST3-H2A2, a synthetic peptide. ST3-H2A2 selectively targets the N-terminal domain of STAT-3 and inhibits dimerization (Wake and Watson, 2015). In preclinical trials, ST3-H2A2 was found to inhibit STAT-3 N-terminal domain, resulting in the blockage of STAT-3 dimerization and apoptosis in prostate cancer cell lines (Timofeeva *et al.*, 2013).

### Strategies to prevent nuclear translocation

The function of stimulated STAT-3 depends on the transfer of homodimers from underneath the plasma membrane into the nucleus (Heinrich *et al.*, 2003; Liu *et al.*, 2005; Frank, 2007; Herrmann *et al.*, 2007; Lui *et al.*, 2007; Quesnelle *et al.*, 2007; Fletcher *et al.*, 2009). Stopping the transfer of STAT-3 dimers *via* the nuclear pore complex can be a strategy to block transcriptional activity of STAT-3 (Liu *et al.*, 2005; Lui *et al.*, 2007). Yet, the thorough process and uniqueness of the mechanisms that facilitate the shifting of STAT-3 from the periphery of the cell to the nucleus has to be clear. Large protein multiplexes perhaps move into the nucleus *via* the nuclear multiplex pore from side to side the nuclear pore composite, in a progression aided by α and β proteins (Liu *et al.*, 2005; Lui *et al.*, 2007). Importin β along with importins α5 and α7 have been involved in the channeling of phosphorylated STAT-3 through the nuclear pore (Liu *et al.*, 2005).

Nonetheless, it is reported that nuclear import of STAT-3 might be self-determining for tyrosine phosphorylation and intervened through importin α3 (Lui *et al.*, 2007). The nucleocytoplasmic transfer of STAT-3 reveals a forceful stable state among proportions of export and import (Liu *et al.*, 2005; Lui *et al.*, 2007). But interestingly, phosphorylated STAT-3 enters more quickly than non-activated STAT-3 (Liu *et al.*, 2005). Inside the nucleus, dephosphorylation of STAT-3 is carried out by the nuclear PTP-TC45, a suitable substrate to facilitated transfer of exportin-1 (Lui *et al.*, 2007). Up to present time, small-molecule inhibitors of importins α3, α5, or α7 have not been recognized. In addition to effects of Karyostatin 1A, a newly recognized importin β inhibitor, have so far to be described with respect to nuclear import of STAT-3 (Liu *et al.*, 2005; Lui *et al.*, 2007). This importin β inhibitor was trialed on HeLa cell lines (Soderholm *et al.*, 2011).

By using Ratjadone A or Leptomycin B, inhibition of exportin 1 hinders nuclear import of STAT-3, as well as diminishing intensities of STAT-3 phosphorylation and STAT-3-facilitated transcription, which leads to increased apoptosis (Liu *et al.*, 2005). Rajtadone-A was tested on N2A (mouse neuroblastoma) cell lines, while Leptomycin B was examined on human lung carcinoma cell lines (A549). In both of these trials, green fluorescence protein encoding marker genes were also transfected along with the inhibitors and then analyzed under the fluorescence microscope (Köster *et al.*, 2003; Rahmani and Dean 2017). The importins have specific NLS (nuclear localization signals) receptors, and these importins are expressed after the stimulation of cytokines. The elements Arg214/215 and Arg414/417 are essential for the binding of STAT-3 dimer with importins.

Artificially designed inhibitor proteins bimax1 and bimax2 have strong affinity for NLS receptors ultimately inhibiting the nuclear translocation of STAT-3 (Kosugi *et al.*, 2008; Ma and Cao 2006). For the assessment of bimax1 and bimax2, designed recombinant vectors (pDs-Red-bimax) were transfected into HeLa cell lines. After that, fluorescent microscopy was used to examine the location of red fluorescent protein. This indicated the successful transfection of bimax1 and bimax2, inhibiting the proliferation of HeLa cells (Wu *et al.*, 2017b). Regardless, inhibition of general trafficking by any small-molecule inhibitor through the nuclear membrane is probably harmful (Liu *et al.*, 2005). In future studies, scientists must design competitive inhibitors of importins. The cytokine stimulus for expression of importins must be blocked, or the elements Arg214/217 and Arg414/417 must be modified to prevent the nuclear translocation of STAT-3 dimer.

### Indirect inhibition of the STAT-3 signaling pathway

The activation of Tyr kinases receptors is linked to the STAT-3 pathway. The transcription of STAT-3 targeted genes in tumor cells can be blocked by EGFR and Src (upstream tyrosine kinase inhibitor). Beside these inhibitors, there are other small molecules that affect upstream tyrosine kinase and hence are indirect inhibitors of the STAT-3 signaling pathway, e.g. JSI-124 (van Kester *et al.*, 2008). This molecule suppresses phosphorylation of STAT-3 in tumor cells of humans and mouse by inhibiting upstream kinase.

Growth retardation and apoptosis in v-Src-transformed mouse fibroblast, lung and colon carcinomas, and lymphoma cell lines is observed by the administration of JSI-124 at a concentration of 10 μM (Huang *et al.*, 2012; Nefedova *et al.*, 2005; Su *et al.*, 2008; Sun *et al.*, 2005; van Kester *et al.*, 2008). Antitumor immune response of dendritic cells is also promoted by this inhibitor (Sun *et al.*, 2005). Other agents that have a similar mechanism of antitumor activity have also been discovered (Levitzki 1992; Liu *et al.*, 2008; Tannin-Spitz *et al.*, 2007). Among these, a natural product available in the NCI DTP repository has the ability to inhibit activation and phosphorylation of STAT-3 selectively, which leads to apoptosis of tumor cells (Zhang *et al.*, 2007).

Studies showed that, although these analogs may be more effective inhibitors of the STAT-3 signaling pathway, their exact mechanism of action is still unknown. Tyrphostins (Ferrajoli *et al.*, 2007), resveratrol (Nam *et al.*, 2005), AG490, WP1066 (Iwamaru *et al.*, 2006; Pardanani *et al.*, 2007) and TG101209 (Kotha *et al.*, 2006), indirubin (Lee *et al.*, 2008; Natarajan and Bright 2002), as well as curcumin (Bharti *et al.*, 2003; Kagialis-Girard *et al.*, 2007) act as inhibitors of tyrosine kinase by modulating STAT-3 signaling. Mechanisms of action of these inhibitors are clearly known. However, the use of these agents as specific STAT-3 inhibitors is limited because of their effects in many signal transduction pathways. Second-generation OPB compounds with more favorable toxicity profiles have been identified and are currently being evaluated in early phase clinical trials. The inhibition of upstream tyrosine kinases has led to downstream abrogation of STAT-3 signaling with antitumor effects in multiple preclinical models, including prostate cancer (Gu *et al.*, 2014).

### Strategies to prevent STAT-3-DNA binding

In order to imitate the action of *cis*-regulatory elements present in genes, double-stranded oligodeoxynucleotides are synthesized. These dsODN inhibit the stimulation of STAT-3 dimers; as a result, gene expression activated by STAT-3 and growth of tumor cells is inhibited (Becker *et al.*, 1998; Sen *et al.*, 2009b; Zhang *et al.*, 2007). STAT-3-specific dsODN are also used in mouse to inhibit expression of STAT-3 responsive genes and ultimately prevent tumor cell growth. In one trial, 3.2 mg/kg of a STAT-3 dsODN decoy was injected intramuscularly in cynomolgus monkeys and no apparent adverse effect was observed, in spite of reduced gene transcription of STAT-3 activated genes.

An experimental study was carried out to check biological effects of STAT-3 dsODN decoy when injected intratumorally in HNSCC patients. Although initial results of this study indicate the inhibition of genes targeted by STAT-3, the decoy degrades rapidly in serum and is amenable when injected systemically. Metabolic stability of the double-stranded oligodeoxynucleotides can be improved by chemical modification, but in case of systemic administration, the successful delivery of chemically modified dsODNs remains a considerable challenge. Peptide aptamers were identified in modified yeast and have the ability to inhibit binding of DNA and STAT-3, but they are not commonly used inhibitors because of metabolic stability and cell permeability.

### STAT-3 pathway oligonucleotides

Abnormal stimulation of STAT-3 in various cancerous cells led to the development of STAT-3 inhibitors as an anti-cancer defense (Chiba, 2016). Novel and promising strategies targeting transcription factors have recently emerged. These include the inhibition of transcription factor gene expression using antisense oligonucleotides, inhibition of the STAT-3-DNA binding domain using decoy oligonucleotides, or post-transcriptional gene-silencing using small interfering RNA (Turkson *et al.*, 2004). The inhibitors of STAT-3 contain decoy oligodeoxynucleotides (ODN), which has a great influence on DBD.

ODN is an oligonucleotide with usually 10–20 base pair sequences that is incorporated in cells. ODN attaches to the STAT-3-DNA binding domain and helps to block their binding with the responsive elements exhibiting transcription factors, and hence prevent the process of transcription. In this way, this phenomenon helps reduce gene expression (Furqan *et al.*, 2013; Palma *et al.*, 2015). An ODN targeting of the STAT-3 DNA-binding domain showed a desired pharmacodynamic effects when injected into head and neck malignancies (Sen *et al.*, 2012). ODNs and ASOs block the STAT-3 DNA-binding domain and STAT mRNA, respectively (Palma *et al.*, 2015).

AZD9150, an antisense oligonucleotide inhibitor of STAT-3, was well-tolerated and demonstrated single-agent antitumor activity against the treatment-refractory lymphomas and NSCLC in a phase I clinical trial (Hong *et al.*, 2015). This compound has since progressed to phase II clinical evaluation. This decoy shows similarity with STAT-3 genes and helps prevent the signaling of STAT-3 by blocking the activation of STAT-3 molecules. By using this approach, the expression of STAT 3 genes was reduced without showing any toxicity (Dutzmann *et al.*, 2015).

However, ODNs and siRNA are unsuitable for systemic administration because of their rapid degradation. Studies have also confirmed the *in vitro* efficacy of other STAT-3 DNA-binding domain inhibitors, including platinum (IV) compounds such as CPA-1, CPA-7, and IS3295 (Turkson *et al.*, 2004). However, these compounds lack specificity to STAT-3, and studies informing on their pharmacology as well as suitable therapeutic doses are lacking. These inhibitors, therefore, enhance apoptosis and retard the growth of cells in several types of human cancers (Wong *et al.*, 2017).

Galiellalactone, obtained from *Galiella rufa*, is another inhibitor of the STAT-3 DNA binding domain. Administration of Galiellalactone in mouse xenograft by intraperitoneal injection helps to generate apoptosis in prostate cancer cells. Galiellalactone also helps to decrease the expression of mRNA and blocks luciferase activity (Hellsten *et al.*, 2008). There are also different types of drugs that bind to STAT-3. These drugs are made up of DNA-binding inhibitors and include various peptide conjugates, peptide aptamers, and metal-chelating compounds (Szelag *et al.*, 2016). First, a small inhibitor named as STA-21 has been identified by the method of virtual screening. This inhibitor suppresses the activity of STAT-3 luciferase and STAT-3 DNA binding in breast cancer (Wake and Watson, 2015).

Another inhibitor is S3I-20, which blocks the STAT-3 DNA binding, promotes apoptosis, and inhibits growth in human breast cancer (Palma *et al.*, 2015). Another small molecule known as InS3–54 has also been identified by virtual screening, and helps retard the transcriptional activity of the STAT-3 DNA-binding domain (Huang *et al.*, 2016). InS3-54A18 suppresses STAT-3 DNA binding and inhibits gene expression of STAT-3 (Huang *et al.*, 2014). It also generates apoptosis and inhibits the survival, migration, and spreading of cancer cells in the body. Hence, InS3-54 is an effective treatment that helps to promote various inhibitors of the STAT-3 DNA binding domain and could be a novel anti-cancer therapy (Huang *et al.*, 2014). Other novel strategies involving the activation of endogenous negative regulators of STAT-3 (SOCS and PTPs) are also being explored, but are still incipient.

## Future perspectives

Various methodologies have been discovered to suppress the action of the STAT-3-DNA binding. In mouse models, different techniques have been developed to influence apoptosis, multiplication of cancer cells, and tumor growth (Thomas *et al.*, 2015). To regulate gene expression, STAT-3-DNA binding is mandatory. Hence, the inhibition of the STAT-3-DNA binding domain will invalidate the function of STAT-3, and therefore will help to block the regulation of gene expression. In B16 cells of murine melanoma, DBD-1, which is a small peptide, identifies the STAT-3-DNA binding domain and shows remarkable apoptosis. Therefore, inhibitors of the STAT-3-DNA-binding domain exhibit various anti-cancer effects (Chai *et al.*, 2016)**.**

Recent studies suggest that various STAT-3 inhibitors, such as natural agents, synthetic products, and ODNs can gain clinical application in the future (Huang *et al.*, 2016). It is important to know the mechanism of STAT-3 to help regulate the various signaling pathways for the recognition of different therapies. However, the suppression of STAT3 is not an effective treatment by itself. For this purpose, different therapies may be developed to block the activity of STAT-3, as well as signaling pathway by inhibiting the proteins and DBD involved in this pathway (Banerjee and Resat 2016). Among all the strategies mentioned above to target STAT-3, the most appropriate is the inhibition of the DNA binding domain of STAT-3, as it will directly inhibit gene expression of the anti-apoptotic protein.

## Conclusion

STAT-3 activation plays a major role in carcinogenesis. The constitutive activation of STAT-3 with malignant transformation has been known for 13 years. Since then, various studies have been carried out, confirming STAT-3 as a cancer drug target, and significant work has focused on the discovery of novel STAT-3 inhibitors. A huge number of STAT-3 inhibitors is known to date, as illustrated in this review. Most inhibitors are at the trial phase and not yet used in clinical practice. The chemo-preventive agents used against cancer cells *in vitro* and (in mouse cancer models) *in vivo* should be relatively non-toxic to normal cells and exhibit significant bioavailability. The current review may be a platform to critically evaluate and analyze the global methodologies for STAT-3 targeting and for the emergence of clinically favorable direct STAT-3 inhibitors as innovative anticancer agents.

## Conflict of Interest

The authors confirm that there is no conflict of interest.

## Author contributions

SA conceived and designed this manuscript, MN formulated and supervised the study, MU, KJ, AB did wrote up of manuscript, MK and FA proofread the manuscript technically. All authors read and approved the final version.
